# The Mechanisms of Tetracycline in Shaping Antibiotic Resistance Gene Dynamics in Earthworm Casts During Vermicomposting

**DOI:** 10.3390/toxics13040273

**Published:** 2025-04-03

**Authors:** Zhonghan Li, Fengxia Yang, Ming Yang, Renkai Yan, Keqiang Zhang

**Affiliations:** 1 Agro-Environmental Protection Institute, Ministry of Agriculture and Rural Affairs, Tianjin 300191, China; 2 Dali, Yunnan, Agro-Ecosystem, National Observation and Research Station, Dali 671004, China; 3 Shiyan Municipal Agricultural Ecological Environment Protection Station, Shiyan 442000, China

**Keywords:** vermicomposting, antibiotic resistance genes, earthworm gut digestion, tetracycline exposure

## Abstract

Earthworm gut digestion plays a crucial role in reducing antibiotic resistance genes (ARGs) during vermicomposting, offering significant potential for controlling ARG dissemination in livestock manure. However, the impact of residual tetracycline antibiotics on this process remains poorly understood. Herein, this study systematically evaluated the impact of tetracycline of three concentrations (0, 10, and 100 mg/kg) on ARG dynamics and microbial community evolution during 35-day vermicomposting of cattle manure. The results demonstrated that earthworm intestinal digestion effectively eliminated over 96% of initial ARG load in raw manure. Noticeably, tetracycline stress significantly enhanced total ARG abundance in the casts (*p* < 0.05), with distinct response patterns observed among different ARG types. Mechanistic analysis revealed that tetracycline potentially enhanced ARG persistence through two pathways: (1) promoting horizontal transfer via mobile genetic elements, and (2) altering gut microbial succession patterns that influence ARG host–microbe relationships. These discoveries contribute to our comprehension of antibiotic interference in vermi-remediation processes and provide insights for optimizing ARG mitigation strategies in contaminated livestock manure.

## 1. Introduction

Livestock farming intensification has led to the production of large amounts of livestock manure, which poses a major environmental challenge while also offering potential as a nutrient resource [1,2]. Effective management and treatment of manure are essential to mitigate its environmental impacts and utilize its benefits [3]. However, the extensive use of antibiotics in animal husbandry has introduced antibiotic residues and antibiotic resistance genes (ARGs) into animal feces, which raised concerns regarding environmental pollution and public health [4,5]. Therefore, it is essential to adopt appropriate management practices to solve livestock and poultry manure problems while minimizing the risks associated with ARGs.

Vermicomposting has emerged as a sustainable and cost-effective bioconversion method, representing a promising approach for the management of organic wastes like livestock manure [6]. In this process, earthworms and their associated microorganisms work together to convert organic wastes into high-quality organic fertilizers [7]. Vermicomposting not only enhances the fertilizer of the manure, but also shows the potential to mitigate pollutants such as ARGs. For instance, cow manure vermicomposting has been shown to improve the product’s fertility while reducing ARGs by 53% [8]. Similarly, in sludge vermicomposting, a 42% reduction in ARGs was observed following the vermicomposting process [9]. Consequently, vermicomposting serves as a double-purpose solution for both organic waste management and ARG mitigation. Furthermore, earthworm gut digestion plays a very crucial role in the vermicomposting process. The substrates are excreted in the form of earthworm casts after the action of gut microorganisms and enzymes and other substances, the composition and properties of which change significantly [10,11]. Concomitantly, ARGs and microorganisms within the earthworm casts also change in this process, thus it is meaningful to explore the alterations in earthworm casts for elucidating the contribution and impact of earthworm gut digestion on ARGs.

Tetracycline, a broad-spectrum antibiotic, is extensively utilized in livestock and poultry farming [12]. Tetracyclines are incompletely absorbed in the livestock gastrointestinal tract, with most of doses typically excreted as parent compounds through urine and feces, increasing the risk of management in manure. Based on previous study, tetracycline heightens the risk of the spread of ARGs during cow manure vermicomposting [8]. In addition, tetracycline may potentially influence the digestive process within the earthworm’s gut [13]. Nevertheless, how this influence affects the transfer of ARGs under tetracycline stress still requires further clarification.

The present study postulated that tetracycline impacts the reduction in ARGs and microbial changes via the gut digestive process in earthworms. To clarify this phenomenon, vermicomposting experiments with different levels of tetracycline added (0, 10, 100 mg/kg) were conducted in a laboratory for 35 days. Real-time quantitative PCR (qPCR) and high-throughput sequencing analyses were performed to (1) explore the dynamics of ARGs during earthworm gut digestion, (2) reveal the alterations in microbial communities within the casts, and (3) discover the influence of tetracycline on the fate of ARGs during earthworm gut digestion, along with its underlying mechanisms. Through the above-mentioned studies, the effects of tetracycline on ARGs were systematically examined from the perspective of earthworm gut digestion, which provides new insights into enhancing the reduction in ARGs during the vermicomposting process.

## 2. Materials and Methods

### 2.1. Cow Manure, Earthworms, and Reagents

Cow manure from a farm in Tianjin was used as a raw material and pre-composted for a period of 15 days prior to the experiment to make it more suitable for earthworm growth. The physicochemical properties of cow dung are listed in Table 1.

The common species of earthworms used for vermicomposting, *Eisenia foetida*, was used in this study. The earthworms were fed for one month using pre-composted cow manure to acclimatize them to the cow manure environment. In the present study, earthworms of appropriate size, healthy growth and with distinct reproductive rings were selected. Prior to the experiment, the earthworms were washed in sterile water and put in a clean beaker with wet paper, and kept out of light for one day to empty the gut. Tetracycline hydrochloride (CAS no: 64-75-5, purity > 99%) was purchased from Shanghai Macklin Biochemical Co., Ltd. (Shanghai, China).

### 2.2. Experiment Program

Three vermicomposting treatments were set up in this study: a control treatment (CK), a low concentration tetracycline treatment (T1, 10 mg/kg), and a high-concentration tetracycline treatment (T2, 100 mg/kg). This test was conducted for 35 days (selected based on 30-day preliminary trials showing complete organic conversion, extended to accommodate batch variability and ensure full compost stabilization) with destructive sampling every 7 days to prevent cross-temporal interference. It also avoided cumulative disturbances from repeated sampling.

The vermicomposting experiment was conducted using black plastic round box (diameter, 15 cm; height, 12 cm). Each box contained 500 g of cow manure. Different concentrations of tetracycline solution were prepared by dissolving the tetracycline in sterile distilled water. The solution was added by stirring while spraying and verified by measuring different samples from the same system to make sure the tetracycline was mixed well. Each box was numbered and labeled according to the different tetracycline concentrations, and 50 earthworms were placed in each box. Once the experiment started, the experimental setup was placed in an environment protected from light. The top of the box was covered with a layer of gauze to prevent earthworms from escaping. Distilled water was sprayed at regular intervals during the experiment to maintain the water content of the cow manure at about 70%.

### 2.3. Sample Collection

Initial cow manure samples were collected at the beginning of the experiment and stored frozen for testing. Earthworm cast samples were also collected on days 7, 14, 21, 35 of the experiment. The earthworms were placed in a beaker containing moistened filter paper and left in a dark environment overnight. The fresh vermicompost was used as cast samples. It was collected into a centrifuge tube and frozen for testing. The samples of cow manure were subjected to DNA extraction using the FastDNA^®^ Soil Rotation kit (MP Biomedicals, LLC, Santa Ana, CA, USA), and the samples of casts were subjected to DNA extraction using the FastDNA^®^ Feces Rotation kit (MP Biomedicals, LLC, Santa Ana, CA, USA). The DNA samples were stored in −80 °C refrigerator pending determination of ARGs and microbial communities.

### 2.4. Quantification of Antibiotic Resistance Genes and Mobile Genetic Elements

In this study, the target genes comprised 24 subtypes of ARGs, 3 predominant mobile genetic elements (MGEs), and the 16S rRNA gene. These included sulfonamide resistance genes (*sul*-ARGs; i.e., *sul*1, *sul*2), macrolide resistance genes (*erm*-ARGs, i.e., *erm*B, *erm*C), tetracycline resistance genes (*tet*-ARGs; i.e., *tet*X, *tet*O, *tet*Q, *tet*M, *tet*W, *tet*L), quinolone resistance genes (*qnr*-ARGs; i.e., *oqx*B, *qnr*B, *qnr*S), streptomycin resistance genes (*str*-ARGs; i.e., *str*A, *str*B, *aad*A), colistin resistance gene (*mcr*-1), *β-lactam* resistance genes (*bla*-ARGs; i.e., *bla*_GES-1_, *bla*_OXA-1_, *bla*_TEM-1_, *bla*_ampC_, *bla*_NDM_), chloramphenicol resistance genes (*chl*-ARGs; i.e., *cfr*, *fex*A), and MGEs (*intI*1, *intI*2, *tra*A). Real-time quantitative PCR (qPCR) analysis was performed using the 7500 real-time fluorescence quantitative PCR instrument (Applied Biosystems, Waltham, MA, USA). To ensure the accuracy and stability of the assay, the DNA samples were diluted ten-fold with ddH_2_O, depending on the concentration of the DNA samples and the quantitative range of the standard curve. Detailed test methods are in the Supporting Information. The qPCR reaction system is depicted in Table S1. For each DNA template, three parallel reactions were set up, with sterile water serving as a negative control. Table S2 contains a list of the PCR primers, PCR settings, and related references. The qPCR detection method in this study followed the protocol in the reference literature [14].

### 2.5. Microbial Community Analysis

The V3–V4 hypervariable region of bacterial 16S rRNA gene was amplified with the universal primer 338F and 806R. Deep sequencing was performed on the Novaseq 6000 (Illumina, Inc., San Diego, CA, USA) platform at Beijing Allwegene Technology Co., Ltd. (Beijing, China) The methodology is described in Supporting Information.

### 2.6. Data Analysis

The mean and standard deviation of the absolute abundance of each type of ARG in the samples were calculated using Microsoft Excel 2021. Bar graphs and heat maps were plotted using Origin 2021. Data were analyzed by an ANOVA using SPSS 27.0 with *p* < 0.05 as the level of significant difference. Network analysis was carried out using Gephi v0.10.1 software.

## 3. Results and Discussion

### 3.1. Distribution of and Variation in Antibiotic Resistance Genes in Vermicomposting

**Figure 1:**
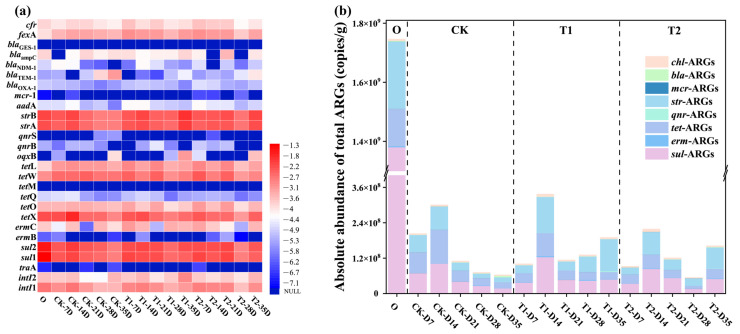
(**a**) Variations in ARGs and MGEs at different time stages during vermicomposting casts. (**b**) The absolute abundance of total ARGs in vermicomposting casts.

In this study, a total of 22 subtypes of ARGs resistant to 8 classes of antibiotics and 3 specific MGEs were detected, as depicted in Figure 1a. Based on the heat map analysis results, both *sul*-ARGs and *str*-ARGs had the highest abundance and detection rates in cow manure and earthworm casts of all stages, with absolute abundances ranging from 10^7^ to 10^9^ copies/g, and 10^7^ to 10^8^ copies/g, respectively. This was followed by *tet*-ARGs, and although *tet*M was not detected, the abundance of *tet*X, *tet*W, and other isoforms were all high, ranging from 10^7^ to 10^8^ copies/g. The reason may be that these antibiotics are widely used due to their low price and good bactericidal effect, leading to the presence of a high abundance of ARGs in the vermicomposting feedstock cow manure and casts [15]. Among them, the abundance of *qnr*-ARGs and *erm*-ARGs was consistently low, particularly in casts. Their absolute abundances were in the range of 10^4^–10^6^ copies/g and 10^3^–10^6^ copies/g, respectively. Another concern is *bla*-ARGs, which were high-risk ARGs for humans, and of which the abundance remained low throughout the vermicomposting process. This is consistent with previous findings [16]. It can be seen that different types of ARGs have different trends in casts at different stages of vermicomposting.

However, in general, the content of ARGs in the casts after digestion by earthworms’ guts was lower than that in the initial cow manure to varying degrees. This indicates that earthworm gut digestion had a significant removal effect on ARGs, which can be more visually observed in Figure 1b. The average abundance of ARGs after gut digestion by earthworms was reduced from 1.75 × 10^9^ copies/g in initial cow manure to 6.33 × 10^7^ copies/g, 96% reduction compared to the initial cow manure. In particular, high concentrations of *sul*-ARGs, *str*-ARGs and *tet*-ARGs in the initial cow manure were all significantly reduced in the casts after digestion in the gut (*p* < 0.05). For *sul*—ARGs, the removal rate was in the range of 90–98%. Meanwhile, *str*-ARGs and *tet*-ARGs also had relatively high removal rates, which were in the range of 47–94% and 11–92%, respectively. It can be seen that earthworm gut digestion has a strong effect on the reduction in these three types of ARGs. It can be seen that the cow manure that passes through the earthworm gut can reduce the amount of ARGs, which is consistent with previous studies [17]. From treatments with different tetracycline concentrations, it was found that the levels of ARGs in casts at different transformation stages under tetracycline pressure were mostly higher than those in the CK group. At the final stage of transformation, the total ARGs in casts increased from 6.33 × 10^7^ copies/g in the CK treated group to 1.90 × 10^8^ copies/g in T1 and 1.62 × 10^8^ copies/g in T2, respectively. Compared with the CK group, the abundance of ARGs in T1 and T2 casts were significantly higher, by 2-fold and 1.56-fold, respectively. This result confirms that tetracycline affects the reduction in ARGs in the earthworm gut.

### 3.2. Fate of Antibiotic Resistance Genes in the Casts Under Tetracycline Stress

**Figure 2:**
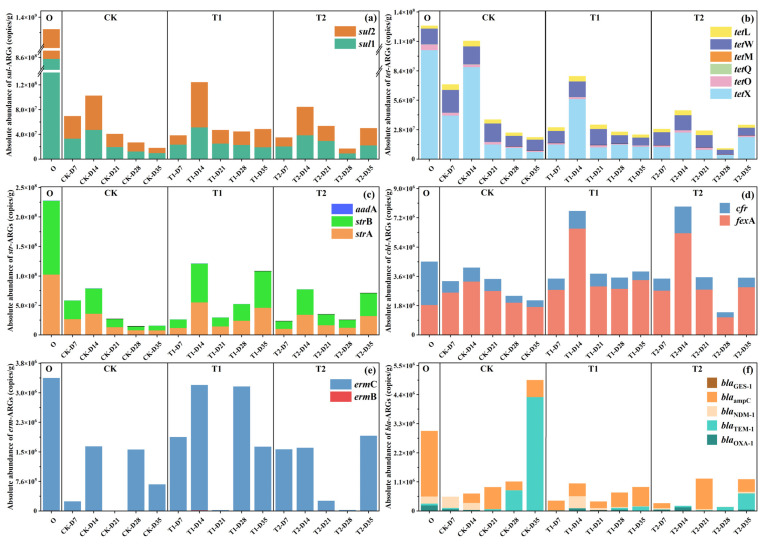
Changes in the absolute abundance of major ARGs in cow manure and casts during vermicomposting: (**a**) *sul*-ARGs, (**b**) *tet*-ARGs, (**c**) *str*-ARGs, (**d**) *chl*-ARGs, (**e**) *erm*-ARGs, and (**f**) *bla*-ARGs.

In order to further understand the changing pattern of each subtype of ARG during the vermicomposting process at different stages after digestion by earthworms in the gut, the bar charts of the changes of different types of ARGs were plotted in this section (Figure 2). Initial cow manure had the highest abundance of *sul*-ARGs at 1.38 × 10^9^ ± 4.12 × 10^7^ copies/g (Figure 2a). The percentage of *sul*1 was even higher at more than 62%. The abundance of *sul*-ARGs in different tetracycline-treated casts at different stages showed a trend of decreasing first, increasing in the middle and finally decreasing significantly (*p* < 0.05). At the end of the transformation, the abundance of *sul*-ARGs in the casts of the CK group was significantly reduced to 1.81 × 10^7^ ± 1.02 × 10^5^ copies/g with a removal rate of 99% (*p* < 0.05). It is evident that gut digestion in vermicomposting has an extremely significant reduction effect on *sul*-ARGs. A previous study found that the abundance of *sul*-ARGs was significantly reduced after vermicomposting [18], which is consistent with the present study. However, with the addition of tetracycline, the abundance in the casts at the end of transformation increased to 4.86 × 10^7^ ± 3.53 × 10^5^ copies/g (T1) and 5.01 × 10^7^ ± 3.19 × 10^5^ copies/g (T2), respectively, which were enhanced by 168% and 177% compared to the control (*p* < 0.05). It is evident that tetracycline may drive the transfer of ARGs hosts in the earthworm gut and affect their reduction of ARGs. The trend of *tet*-ARGs was basically similar to that of *sul*-ARGs, with a significant decrease in *tet*-ARGs abundance in control casts, but its gradual increase with tetracycline content (Figure 2b). However, its increase under tetracycline stress was not as drastic as that of *sul*-ARGs. The results of previous studies on cow manure vermicomposting under tetracycline stress showed that the abundance of *tet*-ARGs in the substrate decreased with increasing tetracycline [8]. This result is contrary to the present study. The possible reason is that tetracycline drives the transfer of ARGs by specific microorganisms in the earthworm gut [19]. In contrast, in the substrate system, the decrease in the abundance of its host microorganisms may be due to the change of its environment or the competition of microorganisms, which reduces the content of *tet*-ARGs [20].

Earthworm gut digestion was also effective in removing *str*-ARGs, showing a trend of decreasing, then increasing, and finally decreasing in the CK treatment (Figure 2c). However, with the addition of tetracycline, during vermicomposting the increase in *str*-ARGs was more obvious. Particularly, in the T1 treatment, the abundance of ARGs in the casts at the end stage of transformation even increased twofold. The trend of *chl*-ARGs is shown in Figure 2d, and the changes in ARGs in the whole process were not as drastic as the rest of ARGs. But similarly, the addition of tetracycline increased their abundance. It is noteworthy that on day 14, the peaks of *chl*-ARGs in the T1 and T2 treatment increased dramatically. It gradually increased with the addition of tetracycline, but still had some removal effect. There was a significant reduction in *cfr* in casts at all stages of each treatment. However, the removal of *fex*A in the CK group was not significant, and with the addition of tetracycline, the abundance of *fex*A increased even above the initial cow manure’s *fex*A content. Interestingly, for *bla*-ARGs, the absolute abundance of *bla*-ARGs increases significantly in the treatment group after transformation by earthworms (*p* < 0.05), with *bla*_TEM-1_ in particular contributing the vast majority of the increase. However, the addition of tetracycline reverses this phenomenon and decreases the amount of *bla*-ARGs in transformed casts. This phenomenon is consistent with previous studies [8], which showed that tetracycline increased the removal of *bla*-ARGs by vermicomposting. The reason for this may be that tetracycline reduced *bla*-ARG abundance through the selective inhibition of susceptible bacterial hosts carrying these genes. This population-level suppression diminished the ecological niche available for *bla*-ARG propagation, thereby decreasing their prevalence in the microbial community, further ultimately reducing their abundance [21].

### 3.3. Changes in Mobile Genetic Elements in Casts Under Tetracycline Stress

**Figure 3:**
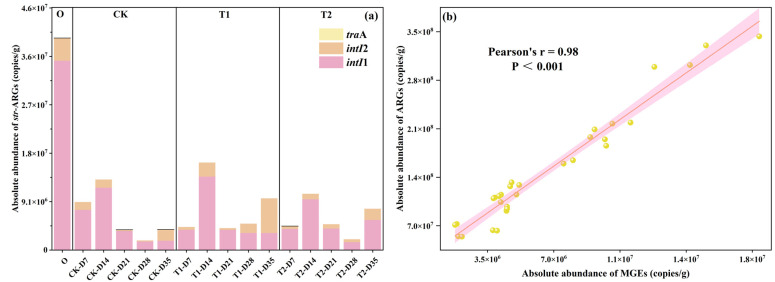
(**a**) Changes in MGEs in casts. (**b**) Correlation analysis of ARGs and MGEs.

Mobile genetic elements (MGEs), acting as transfer vectors for ARGs, enable host microorganisms to uptake exogenous ARGs via horizontal gene transfer (HGT) mechanisms (like conjugation, transduction, and transformation), and integrate them into the bacterial genome. Previous studies have verified that HGT is crucial for ARG spread in the environment [22]. In this study, *intI*1 (class 1 integrase gene), *intI*2 (class 2 integrase gene), and *tra*A (conjugative plasmids) were examined in all samples, and the results are shown in Figure 3a. It was found that *intI*1 and *intI*2 were detected in all samples, while *tra*A was detected in 25%. The absolute abundance of MGEs in the initial cow manure was high at 4.0 × 10^7^ ± 2.96 × 10^5^ copies/g, with *intI*1 accounting for the largest proportion at 89%. The abundance of MGEs in casts was significantly reduced after earthworm transformation (*p* < 0.05), with 90% removal. It can be seen that earthworm gut digestion significantly reduces the fugitive level of MGEs and reduces the risk of HGT in ARGs, which is the same as previous studies [23,24]. The trend of changes in MGEs in the tetracycline treatment group was also approximately the same as that in the CK treatment group. It was also found that the changes in MGEs of all samples were close to the trends of ARGs, while the results of correlation analysis (Figure 3b) showed that there was a highly significant strong positive correlation between ARGs and MGEs (r = 0.98, *p* < 0.001). This further confirmed the previous conclusion that vermicomposting could reduce the abundance of MGEs and the spread of ARGs [25].

The addition of different concentrations of tetracyclines increased MGEs in the casts after gut digestion in earthworms. Compared to the CK treatment, the MGEs in casts at the end of transformation were increased by 150% and 100% for the T1 and T2 treatments, respectively. This phenomenon shows that tetracycline increases the level of MGEs in earthworm-digested casts and increases the risk of spreading ARGs. This is the same as the study on sludge vermicomposting where tetracycline significantly increased MGEs in sludge vermicomposting [13,26]. Although tetracycline increases the amount of MGEs in casts, earthworm transformation under tetracycline pressure still has a good ability to reduce MGEs. Compared to initial cow manure, the abundance of MGEs in casts on day 35 of T1 and T2 treatments was reduced by 76% and 80%, respectively. This result proves that vermicomposting has the potential to treat pollutant-rich cow manure, and it still maintains the ability to reduce ARGs to a certain extent.

### 3.4. Impact of Tetracycline on Bacterial Communities Within Earthworm Casts

**Figure 4:**
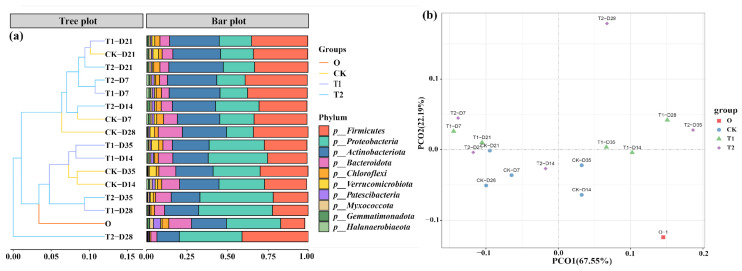
(**a**) Changes in relative abundance at the bacterial phylum level. (**b**) PCoA analysis showing the clustering of communities.

Alpha community diversity in casts before and after gut digestion in earthworms showed significant changes, as shown in Table S3. After intestinal digestion, both Chao1 index and Shannon index in the initial cow manure showed a significant decrease from 6627 ± 367 and 9.56 ± 0.23 to 4511 ± 246 and 7.41 ± 0.12 (*p* < 0.05), respectively. After gut digestion, the richness and diversity of microbial communities showed a decreasing trend. This result is consistent with previous vermicomposting studies [27]. The main reason for this may be that earthworms swallow cow manure, and the rich digestive enzymes in the gut will break down the bacteria. At the same time, the earthworm gut resembles a miniature anaerobic reactor. This environment inhibits the cultivation of dominant aerobic and facultative bacteria in cow manure [28,29]. This leads to a decrease in the abundance and diversity of microorganisms in the casts. However, after the addition of tetracycline, the Chao1 index and Shannon index in the casts increased significantly (*p* < 0.05). This indicates that tetracycline can increase the microbial abundance in the casts of the earthworm transformation process. In addition, we performed the principal coordinate analysis (PCoA) analysis of the microbial communities of the cow manure and casts samples (Figure 4b), and it can be seen that the first and second principal components explained 67.55% and 22.19% of the selected variance, respectively. The distribution of the different sample points shows that all three conditions, before and after digestion in the earthworm gut, different transformation times and tetracycline addition, changed the microbial structure in the samples. Similar results were obtained for casts from previous vermicomposting of earthworm sludge [30].

Moreover, it is imperative to undertake a thorough examination of the alterations in the microbial community present within each sample, the relative abundance at the microbial phylum level is shown in Figure 4a. Proteobacteria, Actinobacteriota Firmicutes, and Bacteroidota were the major bacterial phylum in the initial cow manure samples with a total share of more than 83%. The abundance of Firmicutes increased significantly after the transformation of earthworms, and its percentage increased from 20% to 45% (*p* < 0.05). The reason for this may be that numerous members of the Firmicutes belong to the gut bacteria, leading to an enrichment of Firmicutes in the casts [31]. In addition, the relative abundance of Actinobacteriota increased after gut digestion. Similar phenomena were observed in vermicomposting [32]. The abundance of Proteobacteria decreased significantly after the earthworm gut digestion (*p* < 0.05), and this change rule is consistent with the results of previous studies [33]. However, in the tetracycline treatment, the abundance of Proteobacteria in the casts at the late stage of transformation was significantly increased (*p* < 0.05), which could be responsible for the increase in ARGs.

**Figure 5:**
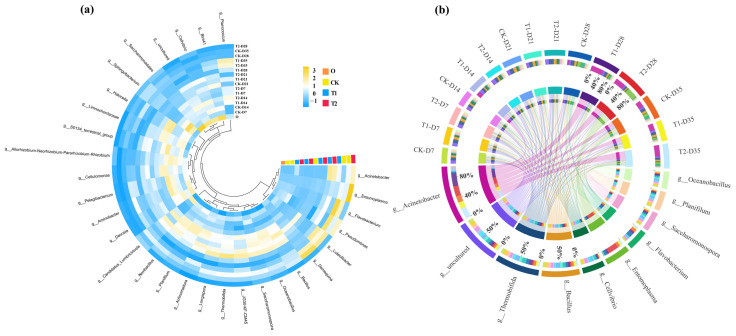
(**a**) Heat maps of the relative abundance of the top 30 bacteria at the genus level in cow manure and different treatment casts (yellow and blue colors indicate high and low abundance of bacteria, respectively). (**b**) Distribution of major bacterial genera in different treatments. Circles represent bacterial phylum and earthworm casts. The upper portion represents the earthworm casts and the lower portion represents the bacterial phylum. The inner lines indicate the connection between the bacterial and the casts. The thickness of the lines indicates the abundance of bacteria.

In order to explore the changes in the bacterial community in more detail, the abundance of bacteria in the first 30 genus is shown in Figure 5a. The bacterial genus with high abundance in the initial cow manure were *Saccharomonospora*, *BIrii41*, *Cellvibrio* and *Devosia*. These genera are common and highly abundant bacterial genera in cow manure, and similar findings have been found in previous studies of cow manure composting [34]. After digestion in the earthworm gut, the dominant genera in the casts changed to *Demequina*, *Luteolibacter,* and *Flavobacterium*. The high abundance of bacterial genera in the gut of earthworms was introduced in the casts, which may also be responsible for influencing the changes in ARGs. The distribution of major bacterial genera in different treatment groups is shown in Figure 5b, whereby tetracycline was found to inhibit the abundance of *Flavobacterium* after gut digestion in earthworms. This suggests that tetracycline has an inhibitory effect on the growth of specific bacteria. Meanwhile, with the addition of tetracycline, there was a general increase in the abundance of *Planococcus* and *Acinetobacter* in the casts. Previous studies of cow manure composting had found that *Planococcus* may be a potential host bacterium for a variety of ARGs [35]. In this respect, tetracycline stress increased the abundance of ARGs after gut digestion of earthworms, which may be related to it.

### 3.5. Mechanisms of Tetracycline’s Influence on Antibiotic Resistance Genes During Earthworm Gut Digestion

**Figure 6:**
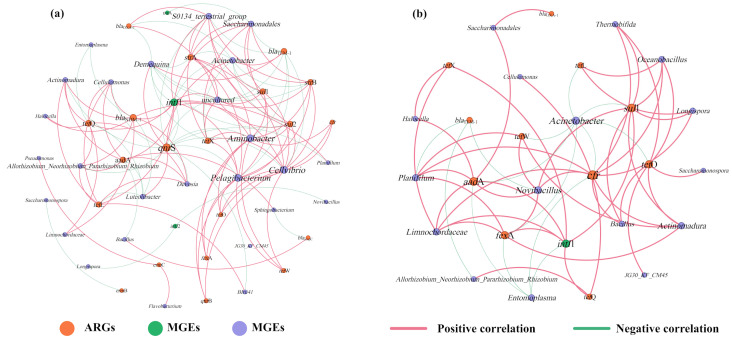
Co-occurrence network analysis of ARGs, MGEs, and microorganisms (genus level) in casts ((**a**) vermicomposting without tetracycline, (**b**) vermicomposting with tetracycline). Connections indicate strong (Spearman’s r > 0.8) and significant (*p* < 0.05) correlations. Node size is proportional to the number of connections, and edge widths are weighted according to the degree of correlation. Pink links indicate positive correlation and green links indicate negative correlation.

To investigate the mechanism of the effect of tetracycline on ARGs in the gut digestive process of earthworms, a co-occurrence network analysis of ARGs, MGEs, and microbial communities was performed here (Figure 6). Previous studies have demonstrated that ARGs significantly and strongly positively correlated with certain bacteria, and this non-random relationship may yield new insights for the study of ARGs and their potential host bacteria [36]. It can be noticed from Figure 6a that the addition of tetracycline changed the structure of the network model. Network plots of casts from vermicomposting treatments without tetracycline showed a strong positive correlation between *Aminobacter*, *Cellvibrio*, *Pelagibacterium*, *Saccharimonadales*, and *S0134_terrestrial_group* with a wide range of ARGs (e.g., *sul*1, *sul*2, *str*A, *str*B, *qnr*B and *tet*W) after gut digestion. In this case, multiple potential host bacteria exist for a class of ARGs. In contrast, with the addition of tetracycline, there was an increase in ARGs shared by the host bacteria and a stronger and closer relationship between them (Figure 6b). At the same time, the potential host bacteria for ARGs changed, shifting to bacteria such as *Novibacillus*, *Planifilum*, *Actinomadura*, *Limnochordaceae,* and *Bacillus*. This demonstrates that tetracycline stress stimulates alterations in ARG potential host microorganisms during the gut digestive processes of earthworms. Similar findings have been reported by previous researchers in sludge vermicomposting. The addition of tetracycline altered the potential host microorganisms of the ARGs [13].

In addition, the relationship between ARGs and potential host microorganisms was more complex in this study without the addition of tetracycline. However, with the addition of tetracycline, the relationship became stronger and more intense. This result is contrary to the results of vermicomposting substrates under tetracycline stress [8]. The reason for this may be that earthworm gut digestion introduces a large number of gut microorganisms and changes the microbial community, but the growth of certain microorganisms is inhibited or promoted in the vermicomposting system, leading to changes in ARGs and host microorganisms [37]. For MGEs, it can be seen that *intI*1 is associated with six microorganisms and four subtypes of ARGs in CK treatment. However, with the involvement of tetracycline, *intI*1 was more strongly associated with microbes and ARGs. Notably, *intI*1 turned out to be strongly and positively associated with high concentrations of *sul*1, which explains the increase in total ARGs after the addition of tetracycline. It is evident that tetracycline also increases the risk of HGT of some of the high-concentration ARGs.

## 4. Conclusions

This study revealed that earthworm gut digestion significantly reduced total ARG abundances in cow manure, demonstrating its intrinsic bioremediation capacity. However, the presence of tetracycline residues increased the abundance of ARGs in casts from the earthworm gut digestive by 65–83%. Mechanistic analysis indicated that tetracycline stress altered the richness and diversity, triggering host bacterial reassignment of ARGs. Crucially, tetracycline simultaneously amplified the horizontal transfer risks of ARGs. These synergistic effects explain the observed ARG persistence under antibiotic pressure, highlighting the need to address residual antibiotic in vermicomposting systems.

## Figures and Tables

**Table 1:** Physicochemical properties of cow manure.

Index	Cow Manure
Water content (%)	75.75 ± 1.31
pH	8.52 ± 0.10
Total nitrogen (%)	1.64 ± 0.30
Total phosphorus (%)	1.42 ± 0.20
Total potassium (%)	1.70 ± 0.30
Nitrate nitrogen (mg/kg)	107.12 ± 5.80
Ammonium nitrogen (mg/kg)	261.92 ± 18.76
Organic matter (%)	52.25 ± 1.62

## Data Availability

The data presented in this study are available on request from the corresponding author due to privacy.

## Supplementary Materials

The following supporting information can be downloaded at: https://www.mdpi.com/article/10.3390/toxics13040273/s1, Table S1: The PCR reaction system; Table S2: The qPCR primers and the PCR parameters used in this study; Table S3: Microbial α-diversity in different treatments.
