# Supplementary material for: The Mechanisms of Tetracycline in Shaping Antibiotic Resistance Gene Dynamics in Earthworm Casts During Vermicomposting
Source: Toxics. 2025 Apr 3;13(4):273. doi: 10.3390/toxics13040273 (PMC12031191; doi:10.3390/toxics13040273)
Supplement: Supplementary file 1 [file toxics-13-00273-s001.zip › toxics-3530787-supplementary.pdf]

SUPPORTING INFORMATION for

The mechanisms of Tetracycline in shaping Antibiotic Resistance Gene dynamics in earthworm casts during vermicomposting

Zhonghan Li [1], Fengxia Yang[1,2]*, Ming Yang[1], Renkai Yan[3], Keqiang Zhang[1,2]*

[1] *Agro-Environmental Protection Institute, Ministry of Agriculture and Rural Affairs, Tianjin 300191, China*

[2] *Dali, Yunnan, Agro-Ecosystem, National Observation and Research Station, Dali 671004, China*

[3]*Shiyan Municipal Agricultural Ecological Environment Protection Station, Shiyan 44200, China*

* Corresponding authors

E-mail address: yangfengxiacomeon@163.com (FX. Yang)

keqiangzhang68@163.com (KQ. Zhang)

Postal address: No. 31 Fukang Road, Nankai District, Tianjin City, Republic people of China

S2 Materials and Methods

S2.5 High-Throughput Sequencing

The V3–V4 hypervariable region of bacterial 16S rRNA gene was amplified with the universal primer 338F (5'-ACTCCTACGGGAGGCAGCAG-3') and 806R (5'-GGACTACNNGGGTATCTAAT-3'). For each sample, an 8-digit barcode sequence was added to the 5' end of the forward and reverse primers (provided by Allwegene Company, Beijing). The PCR was carried out on a ABI 9700 PCR instrument (Applied Biosystems, USA) using 25 μL reaction volumes, containing 12.5 μL 2 × Taq PCR MasterMix (Vazyme Biotech Co., Ltd, China), 3 μL BSA (2 ng/μl), 1 μL forward primer (5 μM), 1 μL reverse primer (5 μM), 2 μL template DNA, and 5.5 μL ddH2O. Cycling parameters were 95 °C for 5 min, followed by 28 cycles of 95 °C for 45 s, 55 °C for 50 s and 72 °C for 45 s with a final extension at 72 °C for 10 min. The PCR products were purified using the Agencourt AMPure XP Kit (Beckman Coulter, Inc., USA). Sequencing libraries were generated using NEB Next Ultra II DNA Library Prep Kit (New England Biolabs, Inc., USA) following the manufacturer's recommendations. The library quality was assessed by Nanodrop 2000 (ThermoFisher Scientific, Inc., USA), Agilent 2100 Bioanalyzer (Agilent Technologies, Inc., USA), and ABI StepOnePlus Real Time PCR System (Applied Biosystems, Inc., USA), successively.

Deep sequencing was performed on Illumina Miseq/Nextseq 2000/Novaseq 6000 (Illumina, Inc., USA) platform at Beijing Allwegene Technology Co., Ltd. After the run, image analysis, base calling, and error estimation were performed using Illumina Analysis Pipeline Version 2.6 (Illumina, Inc., USA).

Table S1. The PCR reaction system.

Reagent	Addition amount
SYBR® Premix Ex Taq TM II	10 μl
Forward primers, 10μM	0.4 μl

Reverse primers, 10μM	0.4 μl
ROX Reference DyeII	0.4 μl
DNA extract	2 μl
RNAase-free	6.8 μl

Table S2. qPCR primers and the PCR parameters used in this study.

Target gene	Primer sequence (5′-3′)	Annealing temp. (°C)	Reference
16S rRNA	F-CGGTGAATACGTTCYCGG<br>R-GGWTACCTTGTTACGACTT	55	Suzuki et al., 2000
*sul*1	F-CGCACCGGAAACATCGCTGCC<br>R-TGAAGTTCCGCCGCAAGGCTG	56	Pei et al., 2006
*sul*2	F-TCCGGTGGAGGCCGGTATCTGG<br>R-CGGGAATGCCATCTGCCTTGAG	60	Pei et al., 2006
*qnr*S	F-GCAAGTTCATTGAACAGGGT<br>R-TCTAAACCGTCGAGTTCGGCG	54	Kim et al., 2009
*oqx*B	F-TCCTGATCTCCATTAACGCCCA<br>R-ACCGGAACCCATCTCGATGC	60	Kim et al., 2009
qnrB	F-CAGATTTYCGCGGCGCAAG<br>R-TTCCCACAGCTCRCAYTTTTC	54	Li et al., 2021
*tet*O	F-ACGGARAGTTTATTGTATACC<br>R-TGGCGTATCTATAATGTTGAC	46	Aminov et al., 2001
*tet*W	F-GAGAGCCTGCTATATGCCAGC<br>R-GGGCGTATCCACAATGTTAAC	60	Aminov et al., 2001
*tet*Q	F-AGAATCTGCTGTTTGCCAGTG<br>R-CGGAGTGTCAATGATATTGCA	60	Aminov et al., 2001
tetL	F- GGTTTTGAAYGTYTCATTACCTGAT<br>R- GATAGCTTTCCATATASAGCTGTTCC	55	You et al., 2012
tetM	F-ACAGAAAGCTTATTATATAAC<br>R-TGGCGTGTCTATGATGTTCAC	55	Aminov et al., 2001
tetX	F-AGCCTTACCAATGGGTGTAAA<br>R-TTCTTACCTTGGACATCCCG	60	Aminov et al., 2001
*erm*B	F-CCGATACCGTTTACGAAATG<br>R-TACTTTGGCGTGTTTCATTC	57	Mu et al., 2015
*erm*C	F-GAAATCGGCTCAGGAAAAG	57	Mu et al., 2015

	R-TAGCAAACCCGTATTCCACG		
*str*A	F- TCAATCCCGACTTCTTACCG<br>R- CACCATGGCAAACAACCATA	60	Walsh et al., 2011
*str*B	F- ATCGCTTTGCAGCTTTGTTT<br>R- ATGATGCAGATCGCCATGTA	60	Walsh et al., 2011
aadA	F- GGTTATCGCCGAAGTATCAACTC<br>R- CACCGTAACCAGCAAATCAATATC	56	Johnson et al., 2013
cfr	F-TGTGCTACAGGCAACATTGGAT<br>R-CAAATACTTGACGGTTGGCTAGAG	55	Wang et al., 2017
*fex*A	F- ATTCTCCCGCAAATAACG<br>R- TCGGCTCAGTAGCATCACG	52	Wang et al., 2017
bla$_{\mathrm{OXA-1}}$	F-TATCTACAGCAGCGCCAGTG<br>R-CGCATCAAATGCCATAAGTG	60	Wang et al., 2015
bla$_{\mathrm{ampC}}$	F-CCTCTTGCTCCACATTTGCT<br>R-ACAACGTTTGCTGTGTGACG	58	Wang et al., 2015
bla$_{\mathrm{TEM-1}}$	F-CATTTTCGTGTCGCCCTTAT<br>R-GGGCGAAAACTCTCAAGGAT	58	Wang et al., 2015
bla$_{\mathrm{NDM}}$	a-CGCCATCCCTGACG-ATCAAA<br>s-CTGAGCACCGCATTAGCCG	59	Luo et al., 2013
bla$_{\mathrm{GES-1}}$	F-ATGGCACGTACTGTGGCTAA<br>R-TGACCGACAGAGGCAACTAAT	56	Yang et al., 2016
intI1	F- GGCTTCGTGATGCCTGCTT<br>R- CATTCCTGGCCGTGGTTCT	55	Luo, Y. et al, 2010
intI2	F- TTATTGCTGGGATTAGGC<br>R- ACGGCTACCCTCTGTTATC	58	Goldstein, C. et al, 2001
TraA	F-<br>AAAGAATTCGAAATTGAGGTAACTTATGAATGC<br>R-CCCAAGCTTCGTTTTATTTCCTGTCAGAG	58	Ghigo, J.M, 2001

Table S3. Microbial α-diversity in different treatments.

Treatment	Chao1 index	Shannon index
O	6627±367	9.56±0.13
CK-D7	5673±117	9.31±0.65
CK-D14	6587±407	9.27±0.15
CK-D21	6474±442	9.11±0.46
CK-D28	5246±324	7.87±0.22
CK-D35	4511±246	7.41±0.12
T1-D7	5353±516	8.73±0.48
T1-D14	6167±485	8.75±0.48
T1-D21	5636±342	8.72±0.68
T1-D28	4986±272	7.07±0.46
T1-D35	6304±375	8.66±0.38
T2-D7	5322±432	8.44±0.14
T2-D14	6421±736	9.12±0.16
T2-D21	6415±363	8.85±0.42
T2-D28	2400±210	5.63±0.10
T2-D35	5295±155	7.45±0.19

The related references

1. Aminov, R. I., Garrigues-Jeanjean, N., Mackie, R. I., 2001. Molecular ecology of tetracycline resistance: development and validation of primers for detection of tetracycline resistance genes encoding ribosomal protection proteins. Appl. Environ. Microbiol. 67(1): 22-32.
2. Cattoir, V., Poirel, L., Rotimi, V., 2007. Multiplex PCR for detection of plasmid-mediated quinolone resistance qnr genes in ESBL-producing enterobacterial isolates. J. Antimicrob. Chemother. 60(2): 394-397.
3. Ghosh, S., Ramsden, S. J., LaPara, T. M., 2009. The role of anaerobic digestion in controlling the release of tetracycline resistance genes and class 1 integrons from municipal wastewater treatment plants. Appl. Microbiol. Biotechnol. 84(4): 791-796.
4. Johnson, E. A., 2013. Monitoring foreign gene incorporation into the plastome of Chlamydomonas reinhardtii by multiplex qPCR. Photosynth. Res. 115(1): 81-87.
5. Kim, H. B., Wang, M., Park, C. H., 2009. oqxAB encoding a multidrug efflux pump in human clinical isolates of Enterobacteriaceae. Antimicrob. agents chemother. 53(8): 3582-3584.
6. Luo, Y., Yang, F., Mathieu, J., 2013. Proliferation of multidrug-resistant New Delhi metallo – β – lactamase genes in municipal wastewater treatment plants in northern China. Environ. Sci. Techn. 1(1): 26-30.
7. Marti, E., Balcázar, J. L., 2013. Real-time PCR assays for quantification of qnr genes in environmental water samples and chicken feces. Appl. Environ. Microbiol. 79(5): 1743-1745.
8. Mu, Q., Li, J., Sun, Y., 2015. Occurrence of sulfonamide-, tetracycline-, plasmid-mediated quinolone-and macrolide-resistance genes in livestock feedlots in Northern China. Environ. Sci. Pollut. Res. 22(9): 6932-6940.

9. Palladino, S., Kay, I. D., Costa, A. M., 2003. Real-time PCR for the rapid detection of vanA and vanB genes. Diagn. Microbiol. Infect. Dis. 45(1): 81-84.
10. Pei, R., Kim, S. C., Carlson, K. H., 2006. Effect of River Landscape on the sediment concentrations of antibiotics and corresponding antibiotic resistance genes (ARG). Water Res. 40(12): 2427-2435.
11. Suzuki, M. T., Taylor, L .T., Delong, E. F., 2000. Quantitative analysis of small-subunit rRNA genes in mixed microbial populations via 5'-nuclease assays. Appl. Environ. Microbiol. 66(11): 4605-4614.
12. Volkmann, H., Schwartz, T., Bischoff, P., 2004. Detection of clinically relevant antibiotic-resistance genes in municipal wastewater using real-time PCR (TaqMan). J. of microbial. methods 56(2): 277-286.
13. Walsh, F., Ingenfeld, A., Zampicolli, M., 2011. Real-time PCR methods for quantitative monitoring of streptomycin and tetracycline resistance genes in agricultural ecosystems. J. of microbial. Meth. 86(2): 150-155.
14. Wang, J. L., Mao, D. Q., Mu, Q. H., Luo, Y., 2015. Fate and proliferation of typical antibiotic resistance genes in five full-scale pharmaceutical wastewater treatment plants. Sci. Total Environ. 526, 366-373.
15. Yang, F. X., Mao, D. Q., Zhou, H., Luo, Y., 2016. Prevalence and fate of carbapenemase genes in a wastewater treatment plant in northern China. PloS one 11(5), e0156383.
16. You, Y., Hilpert, M., Ward, M. J., 2012. Detection of a common and persistent tet(L)-carrying plasmid in chicken-waste-impacted farm soil. Appl. Environ. Microbiol. 78(9): 3203-3213.
17. Yang, F.X.; Mao, D.Q.; Zhou, H.; Luo, Y . Prevalence and fate of carbapenemase genes in a wastewater treatment plant in northern China. PLoS ONE 2016, 11, e0156383.
18. Li, H.; Zheng, X.; Tan, L.; Shao, Z.; Cao, H.; Xu, Y., The vertical migration of

antibiotic-resistant genes and pathogens in soil and vegetables after the application of different fertilizers. Environ Res 2021, 203, 111884.
